# Short- and long-term challenges in crop breeding

**DOI:** 10.1093/nsr/nwab002

**Published:** 2021-01-19

**Authors:** Hong Yu, Jiayang Li

**Affiliations:** Institute of Genetics and Developmental Biology, Innovation Academy for Seed Design, Chinese Academy of Sciences, China; Institute of Genetics and Developmental Biology, Innovation Academy for Seed Design, Chinese Academy of Sciences, China; College of Advanced Agricultural Sciences, University of Chinese Academy of Sciences, China

Agriculture provides the primary energy source and nutrients for humans, and is the key factor determining worldwide population and its basic health status. In the history of agriculture, desirable traits beneficial for farming rather than natural growth have been artificially selected through breeding of new elite varieties. Each successful step in crop breeding, e.g. wild plant domestication, crop introduction and the ‘Green Revolution’, led to a period of rapid population growth. In the last seven decades after the Second World War, the world population has grown from 2.5 to 7 billion. Driven by rapid advances in science and technology, the rate of generating crop varieties is accelerating. Taking China as an example, the annual production of its major staple crop, rice, has increased from ∼57 million tons in 1950 to more than 200 million tons at present. More importantly, in the past two decades, the rice grain quality has also been extensively improved, thanks to the discoveries of underlying complex genetic mechanisms. By the end of this century the world population will reach an estimated 10 to 12 billion, and agricultural productivity needs to increase by at least 50% [1,2]. This imposes serious challenges in crop breeding.

Crops use sunlight, CO_2_ and water as their energy resource and major ingredients to produce final products. The demand of producing more food with fewer inputs never ends. One ongoing strategy is to optimize plant architecture, as exemplified by the dwarfism breeding in the 1960s’ Green Revolution and the ideal plant architecture breeding in the new green revolution, but more efforts are needed for a variety of crops. Several other promising strategies have also been proposed, such as elevating photosynthesis efficiency and manipulating the energy flow and allocation in plants. Accompanying the increased food production, the high input of fresh water, pesticide and fertilizer has become a major threat to sustainable agriculture, stipulating the development of new varieties with high use efficiency of agricultural resources.

The ultimate limitation of food production seems to be the sunlight shining on the arable land, but significant cost reduction in new power systems, together with indoor vertical farming, may break this limitation in the future. New types of crops suitable for indoor systems are thus desirable. Another task is to enhance the taste and nutritional value of crop foods, both of which improve the quality of life. The concept of nutritional values has been extensively expanded, along with ever-growing knowledge of different metabolites, in terms of their biosynthesis pathways in plants and effects on human health. For example, resistant starch (RS) is beneficial in preventing type-2 diabetes mellitus and obesity, and recent advances in understanding its biosynthesis pathway have made it possible to create novel daily foods with high RS varieties.

In the longer term, climate change could be the sword of Damocles in agriculture. The increased CO_2_ concentration, the greenhouse effect and the elevated temperature not only limit crop production by directly affecting growth and development, but also bring more frequent extreme weather conditions, such as extreme heat, typhoons, forest fires, ice-sheet loss and sea level rise that seriously affect farming. Moreover, although climate changes seem to be minor every year, the pattern may change drastically when an unpredicted threshold is reached and major crops may fail to adapt. To face this scenario, we need to breed so-called ‘smart crops’ that are highly resistant to extreme weather conditions and capable of rapid adaptation to climate changes, in addition to their high yield and superior quality. This requires extensive exploration of elite genetic resources and a deeper understanding of the mechanisms underlying plants’ response to biotic and abiotic environmental changes. Smart crops represent novel crop varieties or even non-existing species, beyond the improved varieties of existing crops.

To face these challenges, the Chinese Academy of Sciences has set three goals of crop breeding: (i) to build the capability for decoding precise genetic information, (ii) to develop tools to utilize and rewrite genetic information, and (iii) to achieve smart design and breeding of various crops. A Strategic Priority Research Program ‘Precision seed design and breeding’ has been launched and implemented by the Innovative Academy of Seed Design. This program includes the elucidation of molecular basis and development of innovative technologies for precision seed design, creation of new elite varieties by molecular design, and building-up of their platforms; all aim to meet short-term agricultural challenges and prepare for long-term ones. These challenges are enormous, requiring the continuous efforts of and collaborations among global science communities, farming companies, media, governments and all people living on the Earth.
